# Biogeography and Change among Regional Coral Communities across the Western Indian Ocean

**DOI:** 10.1371/journal.pone.0093385

**Published:** 2014-04-09

**Authors:** Timothy R. McClanahan, Mebrahtu Ateweberhan, Emily S. Darling, Nicholas A. J. Graham, Nyawira A. Muthiga

**Affiliations:** 1 Wildlife Conservation Society, Marine Programs, Bronx, New York, United States of America; 2 Life Sciences, University of Warwick, Coventry, United Kingdom; 3 Earth to Ocean Research Group, Department of Biological Sciences, Simon Fraser University, Burnaby, British Columbia, Canada; 4 ARC Centre of Excellence for Coral Reef Studies, James Cook University, Townsville, Queensland, Australia; Leibniz Center for Tropical Marine Ecology, Germany

## Abstract

Coral reefs are biodiverse ecosystems structured by abiotic and biotic factors operating across many spatial scales. Regional-scale interactions between climate change, biogeography and fisheries management remain poorly understood. Here, we evaluated large-scale patterns of coral communities in the western Indian Ocean after a major coral bleaching event in 1998. We surveyed 291 coral reef sites in 11 countries and over 30° of latitude between 2004 and 2011 to evaluate variations in coral communities post 1998 across gradients in latitude, mainland-island geography and fisheries management. We used linear mixed-effect hierarchical models to assess total coral cover, the abundance of four major coral families (acroporids, faviids, pocilloporids and poritiids), coral genus richness and diversity, and the bleaching susceptibility of the coral communities. We found strong latitudinal and geographic gradients in coral community structure and composition that supports the presence of a high coral cover and diversity area that harbours temperature-sensitive taxa in the northern Mozambique Channel between Tanzania, northern Mozambique and northern Madagascar. Coral communities in the more northern latitudes of Kenya, Seychelles and the Maldives were generally composed of fewer bleaching-tolerant coral taxa and with reduced richness and diversity. There was also evidence for continued declines in the abundance of temperature-sensitive taxa and community change after 2004. While there are limitations of our regional dataset in terms of spatial and temporal replication, these patterns suggest that large-scale interactions between biogeographic factors and strong temperature anomalies influence coral communities while smaller-scale factors, such as the effect of fisheries closures, were weak. The northern Mozambique Channel, while not immune to temperature disturbances, shows continued signs of resistance to climate disturbances and remains a priority for future regional conservation and management actions.

## Introduction

A key challenge for modern conservation science is to determine how climate change, ecology, and human resource use interact to influence ecological resilience and ecosystem services [1], [2], [3]. Specific objectives for coral reef conservation are to identify priority sites of high biodiversity value, connectivity, and resilience that may survive climate change, and to develop appropriate management that ensures the persistence of potentially resilient refugia of biodiversity [3], [4]. Implementing these objectives requires evaluation of regional patterns in environmental and ecological variation, as well as potential for adaptation of taxa and the maintenance of biodiversity following large-scale climatic disturbances. However, these regional and seascape patterns interact, can be complex and sometimes counter-intuitive [5]. Therefore, it is increasingly important to understand how biodiversity responds to climate change across large spatial scales.

The western Indian Ocean (WIO) provides a unique environmental gradient to examine the interactive effects of environmental variation, climate change, connectivity, resilience, and adaptation on marine biodiversity. The region includes tropical and subtropical marine ecosystems influenced by complex currents around the island of Madagascar and the African mainland coastline [6], [7]. Analyses of historical water temperature data reveal strong gradients in mean temperature and variability, which leads to different biodiversity responses to the rate of increase and cumulative anomalies during warming events like the 1998 El Niño Southern Oscillation (ENSO) that caused mass coral bleaching [7], [8], [9], [10]. Environmental variability is strongly affected by regional oceanography, the complex geology of the Eastern African coastline and the position of the island of Madagascar and, to a lesser extent, other smaller islands in the region [11], [12]. For example, the South Equatorial Current (SEC) has low variation in temperature as it moves across the WIO through the southern Seychelles and the Mascarene islands; the leeward (western) side of Madagascar has pockets of water retention that produce warmer temperature distributions and infrequent warm-water skewness (low Degree Heating weeks or months), but also has among the most rapid inter-annual temperature rises [7], [13]. As the SEC turns north and slows into the East African Coastal Current, temperatures become more variable and the northern coastline of Tanzania and Kenya experiences more frequent warm-water skewness (more Degree Heating Months) [7], [13]. This complex oceanography and environmental variability can and has influenced coral reef communities because of their variable tolerance to rapid increases in warm water [7], [9], [14], [15]. Northern Madagascar and Mozambique and the mainland of Tanzania have been identified to harbour high-diversity coral reefs dominated by temperature-sensitive corals [10],[14].

The natural and unique oceanographic gradients in the WIO provide an opportunity to examine patterns of biodiversity and biogeography across different environments and to determine the resilience of coral reefs to temperature anomalies and associated coral bleaching events [10], [16], [17]. Scleractinian reef-building corals are vulnerable foundation taxa that are among the early responders to climate change [18], [19]; different taxa and different environments show variable responses to climate change that can complicate conservation and management prescriptions [6], [20]. Studies of coral communities and their associations with biogeography and human resource use can improve our understanding of coral resilience and identify areas of resistance, recovery, and management needs in a changing climate [21].

Coral reefs in the Indian Ocean are experiencing rapid and large-scale changes, which likely began in the early 1980s due to strong temperature anomalies, the strongest of which was the 1998 El Niño Southern Oscillation (ENSO) event [7], [16], [22], [23]. The greatest coral community changes in response to the 1998 anomaly were documented in the northern-central Indian Ocean [6] and since 1998 there have been smaller yet still intense thermal anomalies and coral bleaching events in parts of the region, including previously less disturbed areas in the southern Indian Ocean [24], [25], [26], [27]. The interaction between thermal anomalies and fishing is expected to further influence coral biodiversity by increasing the dominance of stress-tolerant and opportunistic coral taxa [28], [29], [30]. Nevertheless, a global evaluation of the effects of temperature anomalies on coral reefs in and outside no-take marine reserves failed to find any strong effects of fisheries management preventing the impacts of temperature-driven coral declines, possibly because marine reserves are predominantly located in more temperature susceptible locations [8], [13], [31]. These findings did not account for large-scale biogeographic patterns of latitude or mainland/island environments on biodiversity. Therefore, our objectives were to: (1) evaluate regional biogeographic patterns of coral communities along latitudinal and mainland-island gradients, (2) continue to assess the status of western Indian Ocean coral reefs over the 2004 to 2011 period in terms of abundance, biodiversity, and susceptibility to bleaching, and (3) evaluate the possible impacts of smaller-scale factors, specifically fishing and fisheries closures, on the observed patterns and general reef status.

## Methods

### Field methods

We conducted coral reef surveys throughout the western Indian Ocean (WIO) to develop an extensive database on coral reef communities. All surveys were conducted or led by the authors and used the same methods. Between 2004 and 2011, we surveyed 291 coral reef sites in 11 countries (398 site x year surveys) (Table 1). Research clearance was provided by the respective authorities in each country (see *Ethics Statement* below). The time period between 2004 and 2011 represents recovery from the severe ENSO-driven coral bleaching disturbance in 1998. Although smaller bleaching events occurred in 2004, 2005, 2007, 2008, and 2010, they were limited in spatial scale and did not result in large-scale coral mortality at the study sites included here [7], [22]. All surveys were conducted between November and May during periods of maximum temperature stress. We surveyed coral reefs along the East and South African coastline (Kenya, Tanzania, Mozambique, South Africa) and WIO island nations (Maldives, Seychelles, Comoros, Mayotte, Madagascar, Mauritius, Reunion). Sites were either open to fishing (“fished”) or protected with no-take fisheries closures (i.e., Marine Protected Areas or “closures”). Each year, surveys were conducted haphazardly throughout the region, as it was logistically unfeasible to systematically survey the entire WIO in one year. We found no evidence of biased survey design or site selection by latitude, mainland – island environments or fisheries management (Fig. S1, S2).

**Table 1:** Coral reef surveys throughout the Western Indian Ocean between 2004 and 2011.

	2004		2005		2006		2007		2008		2009		2010		2011	
Country	Fished	Closed	Fished	Closed	Fished	Closed	Fished	Closed	Fished	Closed	Fished	Closed	Fished	Closed	Fished	Closed
Maldives			15													
Kenya	4	4	4	5	3	5	5	5	8	5	11	12	7	6	10	4
Seychelles			14	12					12	9					12	9
Tanzania	2	2	6	3			2	6	5				11	10	8	
Comoros															6	1
Mayotte											7	5	3	2		
Madagascar			1	10			6	4	7		15	6			8	
Mauritius	11	2														
Reunion				6								3		2		
Mozambique			8						6	2	5	3	2	5	7	3
South Africa				6				10								

Countries are ordered from north to south latitudes in the WIO. We found no evidence of biased sampling effort over time across latitude, mainland-island environments or fisheries management, however some sites were only sampled in one type of management and most sites were not sampled through the entire time period (see Fig. S1, S2).

We used two methods to monitor coral reef communities: line-intercept transects and roving observer surveys. Coral community structure and composition was recorded using 9 to 12, 10-m haphazardly placed line-intercept transects (LIT) often at two locations within each site [32]. On each transect, corals underlying the LIT were identified to genus, the continuous length of each coral colony (>3 cm) was measured to the nearest centimeter, and absolute percent coral cover was estimated as the proportion of total length of the coral taxon over the total cover of coral and other benthic categories. In total, we conducted LIT surveys at 119 sites (210 site x year replicates). Sites surveyed with LITs were typically shallow (1–4 m depth, mean ± SD: 3.2±2.4 m), with the exception of 21 sites (n = 63 site x year replicates) surveyed in the Seychelles that were slightly deeper (6.4±1.7 m), but still represented shallow coral reef systems in the region. Coral communities in the Seychelles were recorded using visual estimates within circular point count areas [33], which provided very similar results to LITs [34]. At each site, we estimated total percent hard coral cover and the total percent cover of four major coral families (acroporids, faviids, pocilloporids, and poritiids).

Hard coral communities were also evaluated using roving observer surveys to quantify bleaching susceptibility, coral genera richness and diversity over a larger reef area and typically included deeper sites than the LIT surveys (mean ± SD: 4.6±4.4 m, max 30 m). On each survey, an observer haphazardly delineated ∼20, 2 m^2^ quadrats and within each quadrat, identified every coral colony to genus, and scaled their level of bleaching [6]. We estimated coral generic richness as the number of coral genera that were observed on each roving survey and estimated Simpson's diversity from the proportional abundance (*p*) of colonies within each genus, *i*, for the total number of genera (*S*) within the coral community [35]:
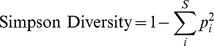



In total, we conducted roving observer surveys at 257 sites (290 site x year replicates). Estimates of percent coral cover from the roving observer surveys were similar to estimates from LITs (*R^2^* = 0.67, *P*<0.0001, *N* = 43 sites where both surveys occurred). We included absolute hard coral cover estimates from both survey types to maximize data coverage across the region; we averaged the values for the 43 sites that had coral cover estimates from both survey methods.

Bleaching susceptibility at each site was estimated from the roving observer surveys as coral community structure weighted by the region-specific sensitivity of each genus [6], [25]. To assess genus-level bleaching sensitivities during each survey, we scored the bleaching intensity, and mortality of each coral colony was assessed on a six-point scale (*c*_0_  =  normal, *c*_1_  =  pale live coral, *c*_2_ = 0–20%, *c*_3_ = >20–50%, *c*_4_ = >50–80%, *c*_5_ = >80–100% of the live coral surface area fully bleached, and *c*_6_  =  recently dead), as detailed in [6]. We then estimated the bleaching response of each coral genus based on a weighted average of bleaching intensity and mortality of the observed colonies within that genus: 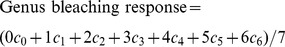



Genus-specific bleaching responses were then averaged across sites to provide a regional bleaching response that was used in the calculation of site susceptibilities. Because surveys occurred during bleaching events and non-bleaching events, we only calculated a genus' regional bleaching responses (BR) from the subset of surveys (N = 141) where the sampling occurred during temperature anomalies and bleaching events (defined as surveys where >10% of coral colonies displayed bleaching). While a 10% cut-off could miss minor bleaching impacts on resistant assemblages, we feel this is a useful cut-off to assess major beaching events for communities. We were then able to calculate bleaching susceptibility at each site based on the relative abundance (RA) of each genus, *i*, and its bleaching response, *BR_i_* [6], [25], where:
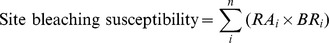



### Data analysis

We evaluated the effects of year (2004 to 2011), geography (latitude, mainland vs. islands), fisheries management (open access vs. no-take fisheries closures), and their two-way interactions on coral community structure and composition. Response variables included: total percent hard coral cover, total percent cover of acroporids, faviids, pocilloporids, and poritiids, coral generic richness and diversity, and bleaching susceptibility. Analyses of richness, diversity, and bleaching susceptibility also included depth and its interactions; depth was not included in the coral cover models that used data from LIT surveys, which mostly occurred on shallow reefs between 0 and 4 m.

We used mixed-effects hierarchical linear models with a random effect of country to take into account the spatially nested nature of our surveys. We started with full models of all main effects and their interactions and carried out model selection using a backwards elimination process by removing non-significant interactions and main effects, confirmed by likelihood ratio tests and AIC scores, until a final model was reached [36]. Model diagnostics were performed visually and the final models met assumptions of normality and homogeneity of residuals (the abundance of faviids and pocilloporids were log_10_+1 transformed to meet assumptions). Finally, we calculated pseudo-*R^2^* and *P*-values of the final model from the relationship between the fitted values and the original observations [36]. We used R [37] for all analyses.

### Ethics statement

Permission for fieldwork was granted from the following agencies: 1. Kenya: National Council of Science and Technology; 2. Mozambique: Eduardo Mondlane University; 3. Mayotte: Head of Equipment, Agriculture and Homing Department; 4. Mauritius: Mauritius Oceanography Institute; 5. Madagascar: Ministère de L^1^Environnement et des Forêts, Direction du Système des Aires Protégées; 6. South Africa: Departments of Science and Technology, the Environmental Affairs and Tourism, Ezemvelo Kwa Zulu Natal Wildlife, and the iSimangaiso Wetlands Park Authority; 7. Seychelles: Seychelles Bureau of Standards and Nature Seychelles; 8. Tanzania: Institute of Marine Science, University of Dar-es-salaam; 9. In the Maldives, we worked with the Banyan Tree Resort who had permit to conduct research; 10. No permit was required for Comoros but we worked with the Coordinator of the Coral Reef Task Force and Focal point of the Nairobi Convention; 11. No permit was required for Reunion. Field studies did not involve manipulation of any endangered or protected species.

## Results

### Coral cover and community composition

**Figure 1:**
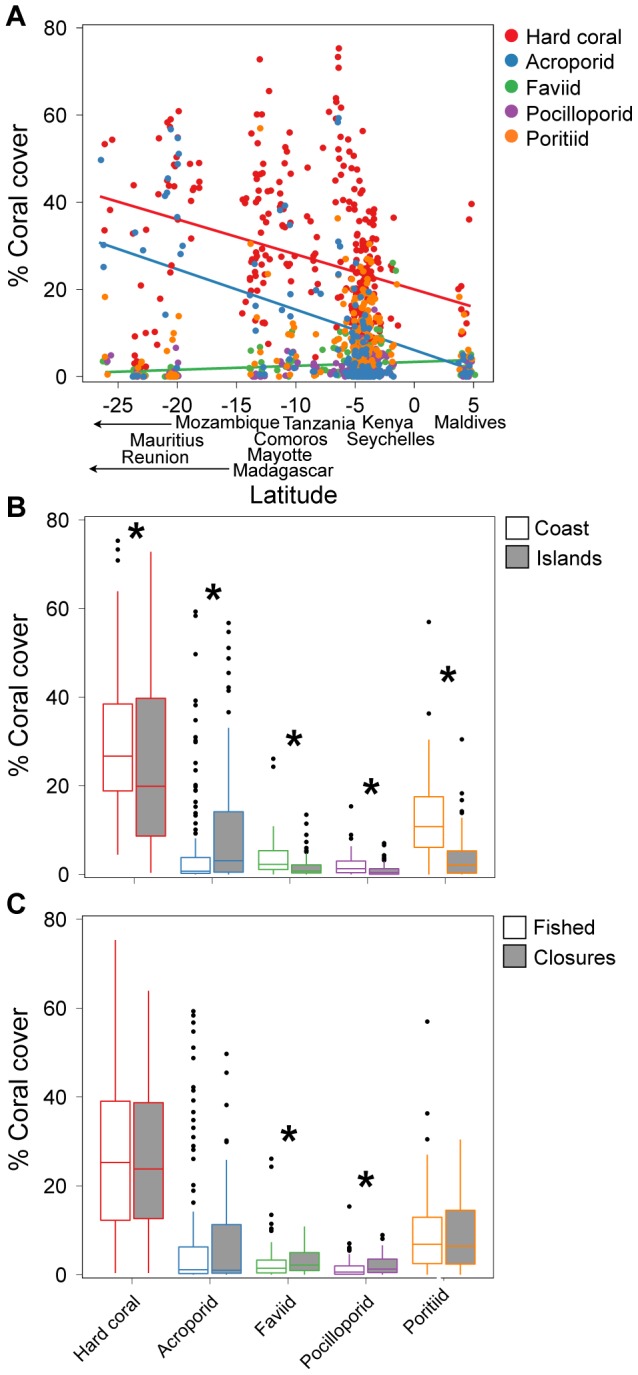
Regional patterns of coral cover and community composition. Coral communities vary across (A) latitude, (B) geography of mainland or island locations, and (C) fisheries management. See Table 2 for model results. In (A), lines indicate significant relationships between coral abundance and latitude. For (B) and (C), asterisks indicate significant differences between groups. Boxplots show median and quartiles, and dots indicate outliers.

There was a significant effect of latitude, mainland-island geography and fisheries management on total live coral cover and community composition of four major taxonomic groups: acroporids, faviids, pocilloporids and poritiids (Table 2; Fig. S3). Total coral and acroporids occurred at higher abundances on southern sites in the WIO while faviids were more abundant at northern sites (Fig. 1A). However, the northern Maldives island sites influenced the observed effect of latitude on hard coral cover (i.e., 15 sites surveyed in 2004). When the Maldives sites were removed from the analysis, total coral cover was no longer influenced by latitude (Table S1).

**Table 2:** Top mixed-effects hierarchical linear models for Western Indian Ocean coral communities.

**Hard coral**	*R^2^*	*P*	**Genera richness**	*R^2^*	*P*
	0.44	<0.0001		0.34	<0.0001
	*t*	*P*		*t*	*P*
Year	0.78	0.434	Depth	-0.14	0.89
Latitude	-2.13	**0.034**	Latitude	6.92	**<0.001**
Geography	-2.59	**0.032**	Depth*Latitude	-2.23	**0.027**
Year*Latitude	2.13	**0.034**			
Year*Geography	2.59	**0.01**			
**Acroporids**	*R^2^*	*P*	**Simpson's diversity**	*R^2^*	*P*
	0.55	<0.0001		0.18	<0.0001
	*t*	*P*		*t*	*P*
Year	-2.85	**0.005**	Depth	2.85	**0.005**
Latitude	-5.50	**<0.001**			
Geography	3.03	**0.029**			
Year*Latitude	5.49	**<0.001**			
Latitude*Geography	4.65	**<0.001**			
**Faviids**	*R^2^*	*P*	**Bleaching susceptibility**	*R^2^*	*P*
	0.21	<0.0001		0.30	<0.0001
	*t*	*P*		*t*	*P*
Latitude	3.42	**0.001**	Latitude	-7.60	**<0.001**
Geography	-1.70	0.15	Management	1.84	0.068
Management	2.74	**0.007**	Latitude*Management	2.35	**0.02**
Geography*Management	-2.36	**0.02**			
**Pocilloporids**	*R^2^*	*P*			
	0.11	<0.0001			
	*t*	*P*			
Geography	-3.7	**0.014**			
Management	2.58	**0.011**			
**Poritiids**	*R^2^*	*P*			
	0.21	<0.0001			
	*t*	*P*			
Geography	-7.29	<0.001			

The original model for each response contained all main effects and their two-way interactions. Geography indicates mainland - island environments. The best-fit top model was reached after step-wise backwards elimination of non-significant predictors; significant parameters for each final model are highlighted in bold.

We observed higher coral cover and more abundant faviids, pocilloporids, and poritiids on mainland East African reefs while acroporids were more abundant in island environments (Fig. 1B). However, the ‘island effect’ on acroporid abundance was influenced by the Maldives; when the Maldives were removed from the analysis, there was no difference in acroporid abundance between mainland and island reefs.

There was higher abundance of faviids and pocilloporids within no-take fisheries closures compared to fished reefs (Fig. 1C). Faviid abundance was also affected by the interaction between fisheries management and geography; faviids were more abundant within coastal closures than closures on islands (islands x management, Table 2).

**Figure 2:**
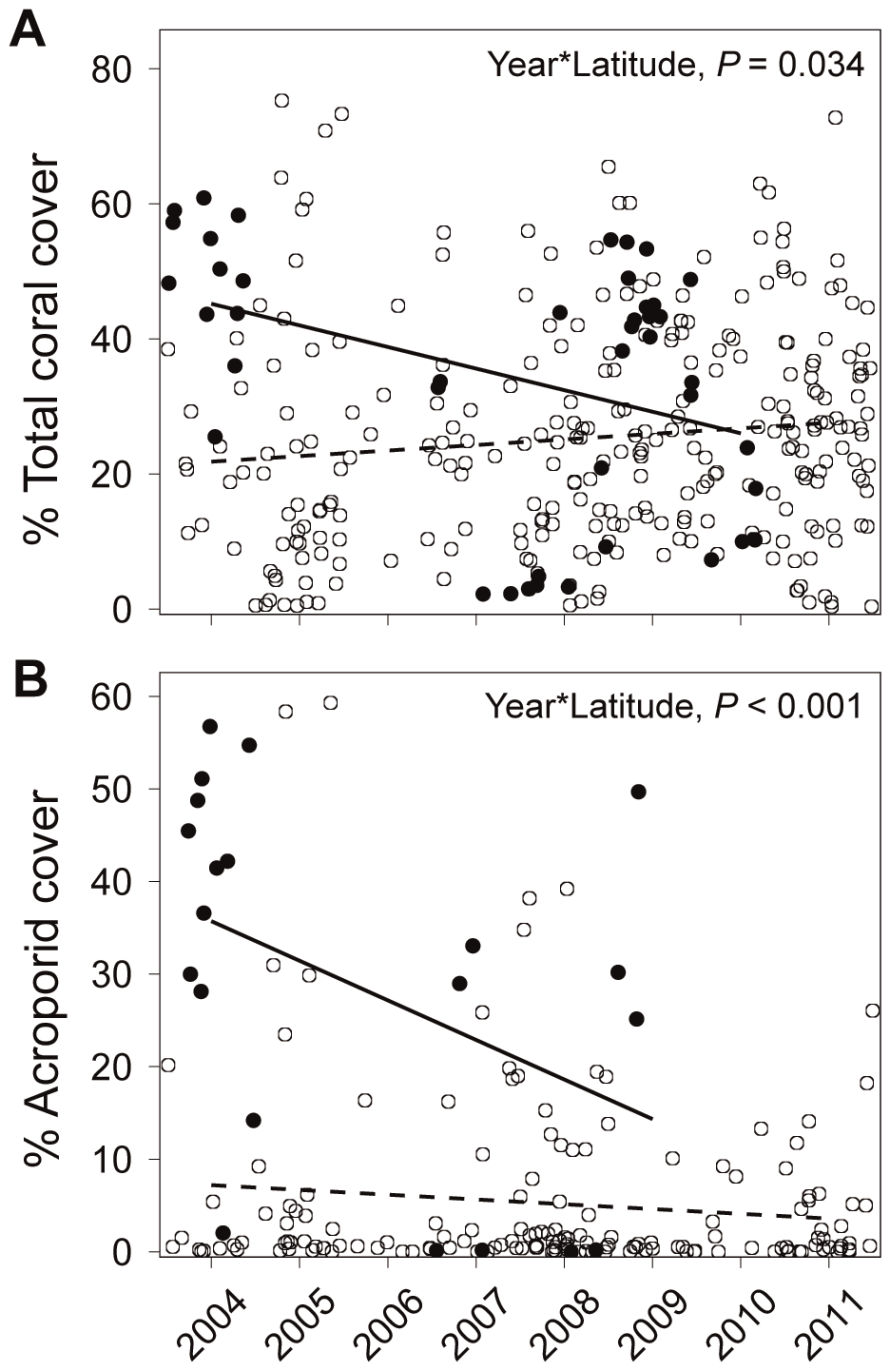
Latitudinal gradients affect coral community change. (A) Total coral cover and (B) acroporid cover are declining faster in the southern WIO (<15°S) than the northern WIO (>15°S). Note: Latitude is shown as ‘north’ (open circles, dashed line) and ‘south’ groups (filled circles, solid line) to illustrate the significant interaction between year x latitude; latitude is a continuous factor is all analyses. See Table 2 for model results.

Coral cover and community composition was largely consistent on sites surveyed between 2004 and 2011. However, there were significant interactions between year and latitude, and year and geography (Table 2). Hard coral cover and acroporids were less abundant on sites surveyed in later years at more southern latitudes (year*latitude interactions, Table 2, Fig. 2). Hard coral cover was more abundant on island environments at later years as opposed to mainland reefs (year*geography, *P* = 0.01) while acroporids were more abundant on island environments at southern latitudes as compared to northern latitudes (latitude*geography, *P*<0.001).

### Richness, diversity and susceptibility to bleaching

**Figure 3:**
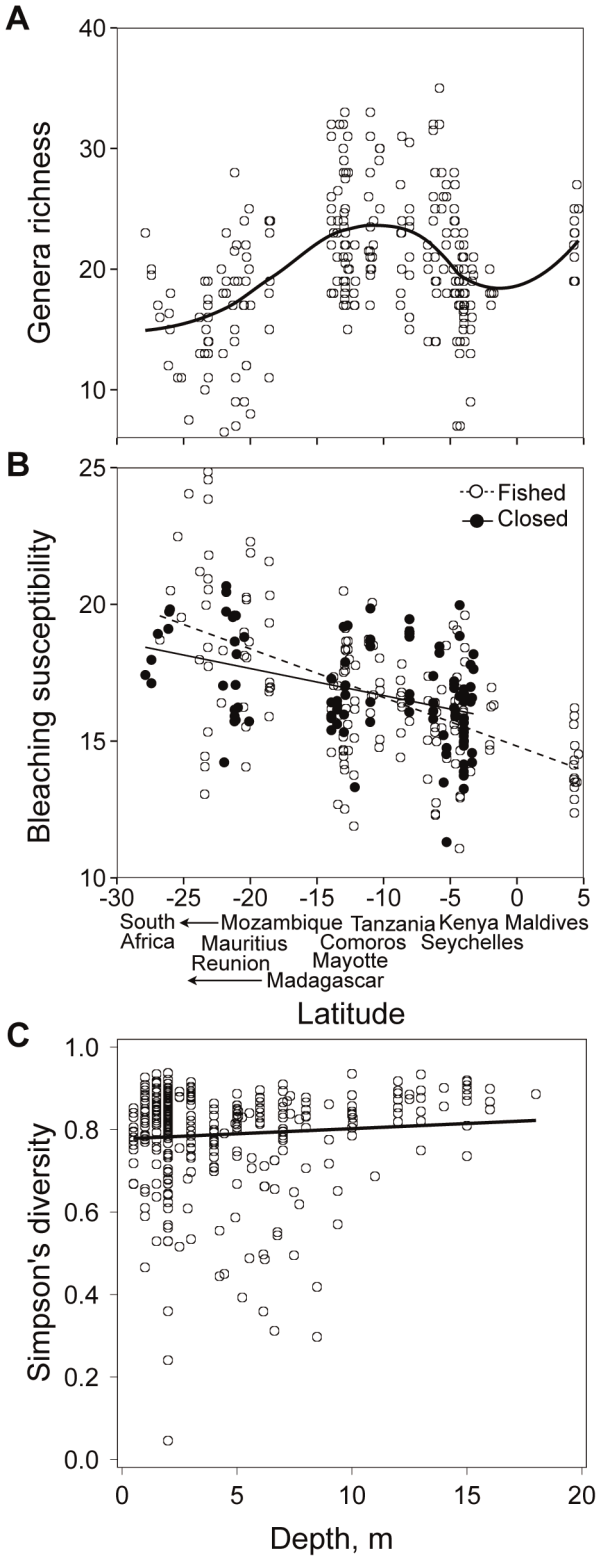
Latitude and depth influence coral richness, diversity and bleaching susceptibility. (A) Coral genera richness peaks at middle latitudes as shown by a locally weighted least squares (LOESS) non-parametric smoother. (B) Bleaching susceptibility of coral communities is higher at more southern latitudes. (C) Simpson's diversity is higher at deeper reefs (up to 30 m) compared to shallower reefs.

Latitude influenced coral generic richness and community susceptibility to bleaching (Table 2). Richness was highest at middle latitudes (∼5°S to 15°S) and increased again at higher northern latitudes in the Maldives (∼5°N) as shown by a locally weighted (LOESS) smoothing function (Fig. 3A). The susceptibility of coral communities to bleaching also increased towards southern latitudes and was influenced by an interaction with fisheries management; coral community susceptibility to bleaching was slightly higher within fisheries closures at northern sites compared to higher susceptibility on fished reefs at more southern sites (Fig. 3B).

Depth also influenced coral generic richness and Simpson's diversity. Deeper sites (up to 30 m) at mid-latitudes contained more coral genera than shallower sites (depth*latitude, *P* = 0.027). Depth also influenced Simpson's diversity; coral communities were more diverse (i.e., even distributions of genera) on deeper sites although there was substantial variation in Simpson's diversity at shallower depths (Fig. 3C).

## Discussion

Coral communities in the western Indian Ocean (WIO) are structured by biogeography and a legacy of past and recent climate-driven coral bleaching events, fisheries extraction, and their interactions. In fact, the large number of significant interactions among these variables indicates the challenges of ascribing the state of WIO corals to a few environmental variables or overarching conclusions. This complexity can challenge conservation and management prioritization and actions focused on corals. Despite some limitations to our regional dataset across space and time (discussed below), we did observe some consistent regional patterns to make inference on some management priorities.

### Biogeography: latitude and mainland-island environments

Latitude was a main biogeographic driver of regional coral communities. Northern coral reef sites in mainland Kenya and Tanzania, and the islands of the Seychelles and Maldives, were greatly affected by the 1998-bleaching event that reduced the abundance of temperature sensitive competitive dominant genera, such as *Acropora* and *Montipora* [6]. We find limited evidence for any large-scale recovery of total coral cover or sensitive acroporid genera after 2004; these taxa remain at low abundances compared to the rest of the WIO region. Northern coral communities are composed of a mix of coral genera, predominantly bleaching-tolerant faviids and poritiids that survived the 1998 bleaching event; any recovery on northern reefs will be site-specific and associated with local environmental factors [22], [29], [38]. However, an important exception is the demonstrated coral recovery in the remote and relatively ‘pristine’ Chagos Archipelago that also occurs at more northern latitudes within the WIO (∼6°S) [6], [7], [39], [40].

The southern WIO and particularly mid-latitude reefs in the northern Mozambican Channel (i.e., western Madagascar, Comoros, Mayotte, northern Mozambique, southern Tanzania) had higher hard coral cover, acroporid cover and subsequently coral communities that are more susceptible to future bleaching. These patterns likely reflect the lesser extent and impacts of temperature anomalies on more southern reefs over the past few decades [8], [16], [22]. Consequently, temperature-sensitive genera, such as acroporids and pocilloporids, remained relatively common in the southern WIO between 2004 and 2011. While not observed here, studies on other reefs have found declines in coral cover and the abundance of acroporids, which probably reflects the effect of small-scale temperature anomalies and reported bleaching in this region since 1998 [26]. Nevertheless, some areas in this region, particularly reefs in southern Tanzania, Madagascar, and northern Mozambique, continue to support healthy populations of bleaching susceptible genera, high coral generic richness and have low to modest levels of environmental stress [10], [14]. Historical temperature studies of this region have found warm but not extreme temperatures (i.e., platykurtic and less skewed temperature distributions), suggesting that temperature stress anomalies are less frequently experienced in these reefs [7], [9], [41]. Furthermore, the northern Mozambique Channel area boasts a unique oceanography of large tides and associated local currents and eddies, and reefs that are less exposed to wave action and storms, which may also explain their high coral richness [13], [14].

Whether reefs were on mainland or islands was an important biogeographic factor structuring coral communities. We found that faviids, pocilloporids and poritiids were more abundant in coral communities on mainland East African coastal reefs [29], [38]. While acroporids have contributed to the recovery of some northern islands in the Chagos and Seychelles archipelagos [39], [40], these bleaching sensitive taxa remain uncommon on disturbed mainland reefs. It should be noted that removal of the Maldives data from the analysis made this effect non-significant for acroporids because the Maldives were dominated by bleaching tolerant massive and sub-massive forms. Coral communities may also depend on the type of substratum; for example, *Montipora* and encrusting faviids have been reported to recover more quickly on granitic than carbonate substrates in the Seychelles [40].

### No-take fisheries closures

Our regional study lends further support to the emerging findings that fisheries closures are not the only solution to maintain coral communities across temperature disturbances [29], [31], [42]. We find no evidence that no-take closures promote more coral genera or more diverse communities, or that closures have promoted the recovery of total coral cover or bleaching-sensitive taxa like acroporids. The main influence of closures was increased abundance of pocilloporids and faviids, although the higher abundance of faviids was limited to coastal reefs (Fig. 1). While fisheries closures can have higher coral cover prior to large coral bleaching events, particularly in older closures, this pattern may be lost or even reversed following a bleaching event [17], [22], [38], [43], [44], [45]. Fisheries closures may also be located in areas with stable background temperatures that promote high coral cover and generic richness that can build up a high abundance of space-occupying and thermally sensitive taxa over time [8], [17], [45], [46], making fisheries closures and their communities more susceptible to rare temperature anomalies that can cause substantial coral bleaching and mortality [13], [29], [31], [43]. While no-take closures are an important management tool for coral reef ecosystem processes and fisheries (see [44]) and may increase recovery rates after disturbances [22], their ability to resist and promote the full community recovery of reef communities to pre-disturbance levels after extreme climate events appears more limited [29], [42]. Nevertheless, the effectiveness of fisheries closures may vary with age, size and compliance and environmental location, all of which requires further investigation [29], [31], [42], [45]. Our study spans a relatively short period of time, and may not capture the full potential of fisheries closures to influence coral recovery rates. Given more time, closures may be able to promote coral recovery, as observed on other remote and ‘pristine’ reefs in Western Australia [47] and the Chagos Archipelago [39]. However, it is concerning that smaller no-take closures within the WIO have not demonstrated full recovery 13 years following the mass coral-bleaching event in 1998. Even if closures eventually promote coral recovery, the window for this potential recovery may be shrinking as climate disturbances become more frequent in the future [18].

### Caveats of a regional approach

While our study combined with previous findings presents a long-term and large-scale perspective for WIO coral reefs, there are cautions and caveats for our conclusions. Our results rely on the site-for-time comparison of this regional approach although logistically our surveys were unable to be fully balanced and carried out at every site in each year. For example, the most northern reefs in the Maldives comprise only fished sites at the start of our study (2004; Table 1); including or not including these sites changed our results (Table S1; see Results). Nevertheless, recent studies of Maldivian reefs, ten years after the 1998 event, reported coral community homogenization that may reflect the uniform and continued influence of the 1998 bleaching event [48]. Further, we were only able to sample Mauritius reefs in early years and surveyed more Mozambican reefs in later years. Additionally, some recovery following the 1998-bleaching event likely occurred at many sites before our regional surveys began in 2004. Smaller and more localized bleaching events affected some reefs throughout our study. For example, we surveyed reefs in Mayotte in 2009 and 2011, before a small-scale bleaching event in late 2011 and early 2012 that would have affected our results. Similarly, Mauritian reefs were badly disturbed by a highly localized bleaching event in 2008, which occurred after our sampling (Moothien-Pillay, K.R. personal communication). It is clear that these space-for-time issues and limitations of our dataset should be taken into consideration when considering the implications of our conclusions. Nevertheless, the objective of our large-scale regional survey (i.e., 398 sites in 11 countries across 30° of latitude over 8 years) was to identify the strongest drivers of biodiversity (and their interactions) as opposed to smaller-scale and more local patterns of coral recovery or community composition.

### Conclusions

Large-scale biogeographic patterns that reflect the importance of geography and local contexts are critical to understand ecosystem recovery from disturbances, such as climatic impacts. While all coral reefs in the WIO are experiencing climate change and coral bleaching, the rate, timing, and magnitude of impacts vary geographically. The northern region is recovering from the devastating 1998 event and, while there was a pulse of recovery shortly after this event [22], the rate appears to have slowed during the 2004−2011 period studied here. Nevertheless, incomplete sampling through the study period in all locations may have also influenced these conclusions. In contrast, there was continued decline in overall coral cover and the abundance of sensitive acroporids in the southern region that may reflect more localized bleaching in the south WIO during this decade.

This study highlights the need to improve efforts and identify actions that can sustain less disturbed reef conditions in the south western Indian Ocean. Specifically, the area between northern Madagascar and the African coastline between northern Mozambique and Tanzania appear to have environmental conditions that promote coral diversity and its persistence [10], [14]. As such, the northern Mozambique Channel is a priority for conservation and management efforts in the WIO. This area continues to persist through climate disturbances compared to other studied locations in the region and consequently it is expected to have the elements of an ecological and possible evolutionary refuge to climate change [10], [14]. It is, however, not immune to climate and human impacts and increasing fishing pressure and gas exploration and extraction are among the current human impacts that could potentially undermine the refuge potential of this area. Appropriate restrictions and management are therefore necessary to help secure the future of these regionally important reefs.

## Supporting Information

Figure S1 **Latitude and mainland-island location are robust indicators of biogeography.** There was no evidence of a sampling bias over time across (A) latitude, however we did find evidence of a sampling bias across (B) longitude, which was removed from further analysis. (C) Mainland-island comparisons were not biased across sampling year. White bars indicate mainland reefs (Kenya, Tanzania, Mozambique, South Africa) and grey bars indicate islands reefs (Maldives, Seychelles, Comoros, Mayotte, Madagascar, Mauritius, Reunion). We use latitude and geography (mainland vs. islands) as robust indicators of regional biogeography in all analyses.
(DOCX)

Figure S2 **Fisheries management sampling across time.** We found no evidence of a sampling bias over time across fisheries management. White bars indicate fished reefs open to exploitation and grey bars indicate sites within no-take fisheries closures.
(DOCX)

Figure S3 **Country-level effects of mainland-island geography and management.** Total hard coral cover varies across countries to reflect latitudinal patterns of biogeography that are also influenced by reef geography and fisheries management.
(DOCX)

Table S1 **Sensitivity analysis of regional trends without the Maldives.**
(DOCX)
