# Supplementary material for: Biogeography and Change among Regional Coral Communities across the Western Indian Ocean
Source: PLoS One. 2014 Apr 9;9(4):e93385. doi: 10.1371/journal.pone.0093385 (PMC3981710; doi:10.1371/journal.pone.0093385)
Supplement: Figure S1 — Latitude and mainland-island location are robust indicators of biogeography. There was no evidence of a sampling bias over time across (A) latitude, however we did find evidence of a sampling bias across (B) longitude, which was removed from further analysis. (C) Mainland-island comparisons were not biased across sampling year. White bars indicate mainland reefs (Kenya, Tanzania, Mozambique, South Africa) and grey bars indicate islands reefs (Maldives, Seychelles, Comoros, Mayotte, Madagascar, Mauritius, Reunion). We use latitude and geography (mainland vs. islands) as robust indicators of regional biogeography in all analyses. (DOCX) [file pone.0093385.s001.docx]

**
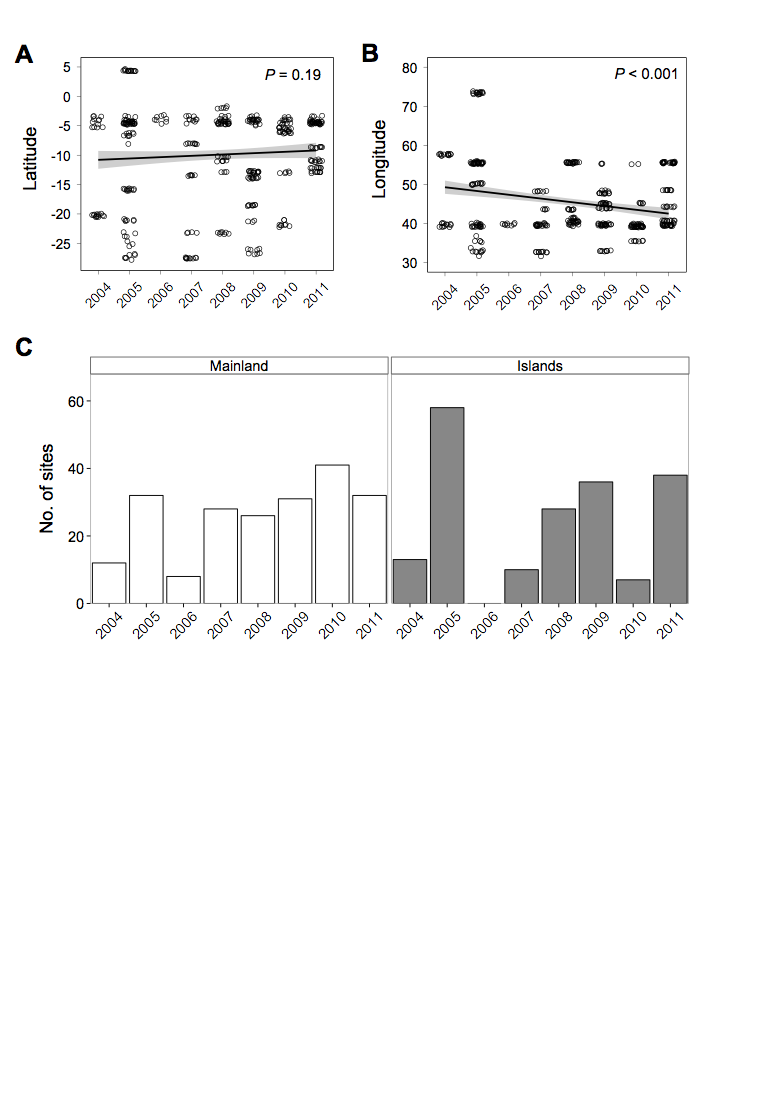
**

**Figure S1. Latitude and mainland-island location are robust indicators of biogeography.** There was no evidence of a sampling bias over time across (A) latitude, however we did find evidence of a sampling bias across (B) longitude, which was removed from further analysis. (C) Mainland-island comparisons were not biased across sampling year. White bars indicate mainland reefs (Kenya, Tanzania, Mozambique, South Africa) and grey bars indicate islands reefs (Maldives, Seychelles, Comoros, Mayotte, Madagascar, Mauritius, Reunion). We use latitude and geography (mainland vs. islands) as robust indicators of regional biogeography in all analyses.
