# Supplementary material for: Biogeography and Change among Regional Coral Communities across the Western Indian Ocean
Source: PLoS One. 2014 Apr 9;9(4):e93385. doi: 10.1371/journal.pone.0093385 (PMC3981710; doi:10.1371/journal.pone.0093385)
Supplement: Figure S2 — Fisheries management sampling across time. We found no evidence of a sampling bias over time across fisheries management. White bars indicate fished reefs open to exploitation and grey bars indicate sites within no-take fisheries closures. (DOCX) [file pone.0093385.s002.docx]

**
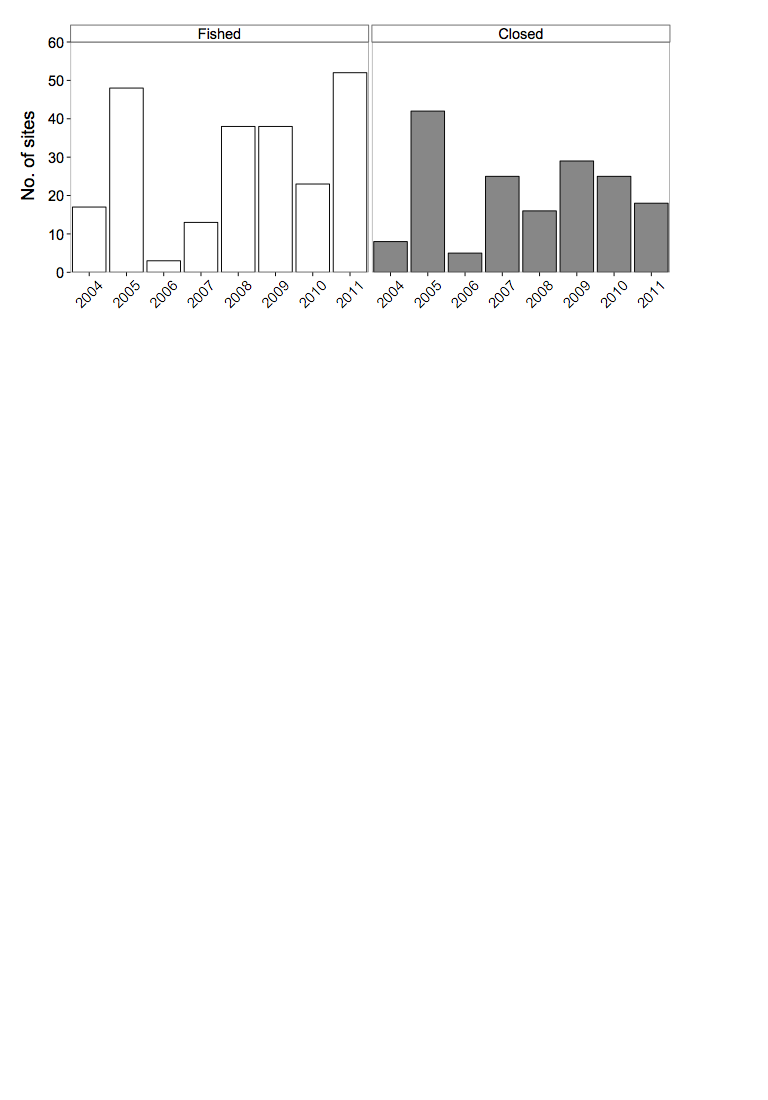
**

**Figure S2. Fisheries management sampling across time.** We found no evidence of a sampling bias over time across fisheries management. White bars indicate fished reefs open to exploitation and grey bars indicate sites within no-take fisheries closures.
