# Supplementary material for: Biogeography and Change among Regional Coral Communities across the Western Indian Ocean
Source: PLoS One. 2014 Apr 9;9(4):e93385. doi: 10.1371/journal.pone.0093385 (PMC3981710; doi:10.1371/journal.pone.0093385)
Supplement: Figure S3 — Country-level effects of mainland-island geography and management. Total hard coral cover varies across countries to reflect latitudinal patterns of biogeography that are also influenced by reef geography and fisheries management. (DOCX) [file pone.0093385.s003.docx]

**
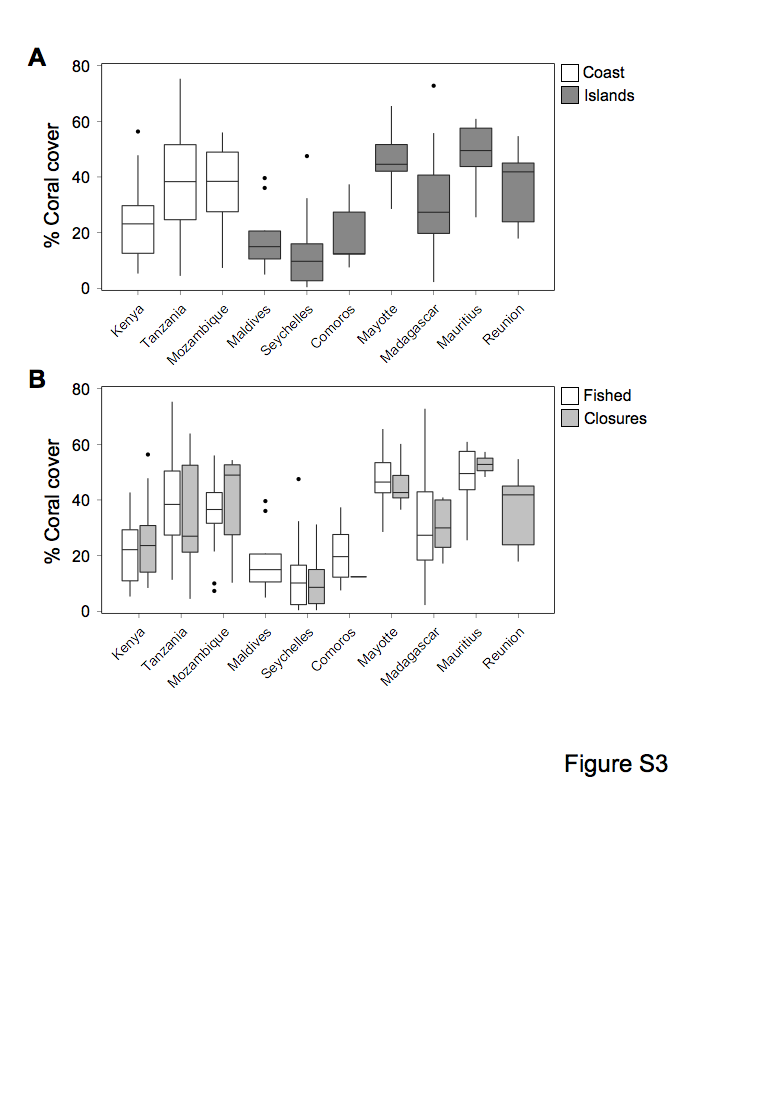
**

**Figure S3. Country-level effects of mainland-island geography and management.** Total hard coral cover varies across countries to reflect latitudinal patterns of biogeography that are also influenced by reef geography and fisheries management.
