# Supplementary material for: Biogeography and Change among Regional Coral Communities across the Western Indian Ocean
Source: PLoS One. 2014 Apr 9;9(4):e93385. doi: 10.1371/journal.pone.0093385 (PMC3981710; doi:10.1371/journal.pone.0093385)
Supplement: Table S1 — Sensitivity analysis of regional trends without the Maldives. (DOCX) [file pone.0093385.s004.docx]

**Table S1. Sensitivity analysis of regional trends without the Maldives.**

| **Hard coral** | *R^2^* | *P* |  | **Pocilloporids** | *R^2^* | *P* |
| --- | --- | --- | --- | --- | --- | --- |
|  | 0.44 | <0.0001 |  |  | 0.11 | <0.0001 |
|  | *t* | *P* |  |  | *t* | *P* |
| Year | -1.48 | 0.139 |  | Geography | -3.74 | **0.02** |
| Latitude^1^ | 1.84 | 0.067 |  | Management | 2.65 | **0.009** |
| Geography | -2.56 | **0.037** |  |  |  |  |
| Year*Geography | 2.57 | **0.01** |  | **Poritiids** | *R^2^* | *P* |
|  |  |  |  |  | 0.21 | <0.0001 |
| **Acroporids** | *R^2^* | *P* |  |  | *t* | *P* |
|  | 0.55 | <0.0001 |  | Geography | -5.91 | **0.004** |
|  | *t* | *P* |  |  |  |  |
| Year | 2.82 | **0.005** |  | **Genera richness** | *R^2^* | *P* |
| Latitude | -5.34 | **<0.001** |  |  | 0.35 | <0.0001 |
| Geography^2^ | 2.06 | 0.108 |  |  | *t* | *P* |
| Year*Latitude | 5.34 | **<0.001** |  | Depth | -0.19 | 0.85 |
| Latitude*Geography | 4.16 | **<0.001** |  | Latitude | 6.89 | **<0.001** |
|  |  |  |  | Depth*Latitude | -2.24 | **0.026** |
| **Faviids** | *R^2^* | *P* |  |  |  |  |
|  | 0.21 | <0.0001 |  | **Simpson's diversity** | *R^2^* | *P* |
|  | *t* | *P* |  |  | 0.18 | <0.0001 |
| Latitude | 2.58 | **0.011** |  |  | *t* | *P* |
| Geography | -1.48 | 0.214 |  | Depth | 3.12 | **0.002** |
| Management | 2.72 | **0.007** |  |  |  |  |
| Geography*Management | -2.24 | **0.027** |  | **Bleaching susceptibility** | *R^2^* | *P* |
|  |  |  |  |  | 0.30 | <0.0001 |
|  |  |  |  |  | *t* | *P* |
|  |  |  |  | Latitude | -6.23 | **0** |
|  |  |  |  | Management^3^ | 1.80 | **<0.001** |
|  |  |  |  | Latitude*Management | 2.28 | **0.023** |

We conducted all analyses without the Maldives due to concern that these 15 northern sites were only surveyed in 2004. Removing in the Maldives affected some of our final models or conclusions, which we now state in the manuscript.

^1^There is no longer an effect of latitude or year*latitude on hard coral cover.

^2^Mainland-island geography no longer affects acroporid cover.

^3^We now observe a significant effect of management on bleaching susceptibility.
